# Biodegradation Assessment of Bioplastic Carrier Bags Under Industrial-Scale Composting Conditions

**DOI:** 10.3390/polym16243450

**Published:** 2024-12-10

**Authors:** Mária Mörtl, Mariem Damak, Miklós Gulyás, Zsolt István Varga, György Fekete, Tamás Kurusta, Ádám Rácz, András Székács, László Aleksza

**Affiliations:** 1 Institute of Environmental Sciences, Hungarian University of Agriculture and Life Sciences, Páter Károly u. 1, H-2100 Gödöllő, Hungary; mortl.maria@uni-mate.hu (M.M.); damak.mariem@phd.uni-mate.hu (M.D.); gulyas.miklos@uni-mate.hu (M.G.); varga.zsolt.istvan@uni-mate.hu (Z.I.V.); fekete.gyorgy@uni-mate.hu (G.F.); 2 Profikomp Environmental Technologies Inc., Kühne Ede u. 7, H-2100 Gödöllő, Hungary; 3 Institute of Raw Material Preparation and Environmental Processing, University of Miskolc, Egyetem út 1, H-3515 Miskolc, Hungary; tamas.kurusta@uni-miskolc.hu (T.K.); adam.racz@uni-miskolc.hu (Á.R.)

**Keywords:** PBAT, biopolymer, composting, biodegradation, FTIR, GC-MS

## Abstract

In recent years, the environmental impacts of plastic production and consumption have become increasingly significant, particularly due to their petroleum-based origins and the substantial waste management challenges they pose. Currently, global plastic waste production has reached 413.8 million metric tons across 192 countries, contributing notably to greenhouse gas emissions. Bioplastics have emerged as eco-friendly alternatives, with bioplastic carrier bags composed of 20% starch, 10% additives, and 70% polybutylene adipate terephthalate (PBAT) being the focus of this research. This study aimed to evaluate the biodegradation of these bioplastic bags under industrial composting conditions, addressing the gap in the existing literature that often lacks real-world applicability. A large-scale composting experiment was conducted using 37.5 tons of manure/wood and 50 tons of biopolymer bags over 12 weeks. Results showed that compost temperatures peaked at 70 °C and remained above 50 °C, pH levels stabilized at 8.16, and electrical conductivity was recorded at 1251 μs cm^−1^. Significant changes were observed in key metrics, such as the carbon-to-nitrogen ratio and organic matter content. Disintegration tests revealed that 95% of the bags disintegrated by the 12th week, though ecotoxicity tests indicated varying germination inhibition rates. Advanced analytical methods (Fourier transform infrared spectroscopy, gas chromatography coupled with mass spectrometry) highlighted morphological and chemical transformations in the bags. This research enhances understanding of bioplastic degradation in real-world composting environments and suggests potential improvements to existing standards, promoting sustainable waste management solutions.

## 1. Introduction

Plastics consist mainly of synthetic polymer compounds derived mostly from petrochemical sources. These compounds are characterized by their high molecular mass, inert, hydrophobic, and long-chain polymers, and their resistance to biodegradation in nature [1]. Inadequate disposal of plastic waste has been linked with various negative environmental impacts. Global plastic waste production reached 413.8 million metric tons across 192 countries in 2023 [2]. In 2022, an estimated 21 million tons of macroplastics leaked into the environment globally, over 30% more than during the previous decade, and plastics are estimated to contribute 3.8% of total global greenhouse gas emissions [3]. Consequently, plastic pollution has now been identified in all major ocean basins, beaches, rivers, lakes, terrestrial habitats, and even isolated regions such as the Antarctic and Arctic regions [4]. Furthermore, plastics are a significant contributor to greenhouse gas (GHG) emissions throughout their life-cycle. According to the Organization for Economic Co-operation and Development [4], 1.8 billion tons of GHGs were emitted in 2019 in relation to the plastic industry, where 90% of these emissions resulted from the production of plastics and their conversion from fossil fuels. These emissions are also linked with detrimental health impacts on humans, such as by the emission of particulate matter [5]. Additionally, GHGs are emitted at the end-of-life of plastics during waste management or incineration and littering. The latter is well known to pose a significant threat not only to the marine environment but also to the safety of animals and humans through the bioaccumulation and toxicity of microplastics in the food chain [6,7,8,9,10,11].

Bioplastics or biopolymers, advertised as environmentally friendly and sustainable materials, have emerged as a viable alternative to conventional plastics. According to European Bioplastics, biopolymers may be divided into three types depending on their resources and biodegradability: (1) bio-based and biodegradable, (2) bio-based but not biodegradable, and (3) biodegradable plastics that do not derive from renewable energy sources [12]. Bio-based polymers derived from renewable resources are created by extraction from biomass molecules (such as sugars found in plants) and then polymerized to create either a direct substitute for an existing plastic type, such as polyethylene, or a unique polymer, such as polyhydroxyalkanoate [13]. Non-synthetic natural polymers are typically starch, natural rubber, and proteins that may be extracted from biomass of different plants, including rice, corn, tapioca, potatoes, soybeans, wood cellulose, wheat fiber, and bagasse [14,15].

On the other hand, synthetic fossil-based biodegradable polymers are derived essentially from crude oil but also from coal and natural gas. Degradability is obtained in these polymers by including unstable linkages (ether, amide, or ester bonds) that are vulnerable to hydrolytic attack [16]. The most important and widely produced fossil-based biodegradable biopolymers are polybutylene adipate terephthalate (PBAT) and polycaprolactone [12].

The biodegradation of biopolymers is a process of mineralization of organic materials by various types of microorganisms (e.g., bacteria, fungi, and archaea). The process involves three main steps [17], starting with the deterioration of the matrix, which consists of the alteration of the mechanical, chemical, and physical characteristics of a polymer. In nature, this process is induced by heat activation, hydrolysis, biological activity, oxidation, photolysis, or radiolysis [18]. The second step of the biodegradation process is the bio-fragmentation step, during which the conversion of polymers by the action of microorganisms to oligomers and monomers occurs. Bio-fragmentation occurs extracellularly, where extracellular enzymes’ active sites form complexes with the substrate and cleave a portion of it off [19]. Finally, the microbial assimilation of the products of the second step takes place. At this point, the microorganisms are supplied with carbon, energy, and nutritional supplies and convert the biopolymers to water, CO_2_, and biomass.

The biodegradation of biopolymers is highly affected by the surrounding environmental factors, as they influence the microbial population and the activity of the individual microorganisms themselves. Humidity, temperature, pH, salinity, the presence or absence of oxygen, and the availability of various nutrients all have a substantial impact on the microbial breakdown of polymers [20]. The process is also affected by the chemical and physical properties of the polymer, including diffusivity, porosity, morphology, cross-linking, purity, chemical reactivity, mechanical strength, thermal tolerance, and electromagnetic radiation resistance [18].

At their end-of-life, biopolymers provide a variety of waste management methods that can generate added value, contributing thereafter to the objectives of a circular economy. Due to their biodegradability, biopolymers such as PBAT can be recycled in some cases [21] or treated as organic waste and can be composted or discarded in anaerobic digestion factories for biogas production [22].

Composting is a controlled, aerobic process that converts organic materials into a nutrient-rich soil amendment and plant fertilizer. The process is mediated by a consortium of microorganisms whose metabolism depends on a variety of environmental conditions mentioned above, with high importance on the carbon-to-nitrogen (C/N) ratio and the characteristics of the input raw materials [23]. The process can be separated into three main phases: the mesophilic, the thermophilic, and the maturation phases. The mesophilic phase is carried out by mesophilic microorganisms that thrive under moderate temperatures (up to 40 °C) where simple organic compounds such as sugars are mineralized. Exothermic reactions are generated by metabolic activity, raising the composting temperature to about 65 °C [24]. At this stage, the mesophilic microorganisms are exhausted, and the composting pile is colonized by thermophilic microorganisms, marking the second phase of the composting process. The thermophilic stage is the most important phase in composting as elevated temperatures in the compost pile kill pathogenic organisms and weed seeds; moreover, microbes break down complex molecules such as proteins, fats, and complex carbohydrates, including cellulose and hemicellulose [25]. As the energy supply in the compost pile depletes, mesophilic microorganisms recolonize the composting pile in the last phase as the temperature falls below 40 °C. In this stage, the remaining carbohydrates, cellulose, and hemicellulose, are broken down by the microorganisms, and the precursors of humic substances are formed.

Biopolymers can be biodegraded in composting facilities combined with other types of organic waste only if they are certified according to the standards listed below in Table 1.

The European standard EN 13432 [26] provides specific requirements for determining the compostability and anaerobic treatability of packaging and packaging materials by addressing four characteristics:

(1) Biodegradability: Referring namely to the conversion of organic materials to CO_2_ by microorganisms. The standard includes a required biodegradation criterion of at least 90% that must be attained in fewer than 6 months (laboratory test method EN 14046 [39]);
(2) Disintegration during biological treatment: Tested through the fragmentation and loss of visibility in the final compost, which is determined in a pilot composting test (EN 14045 [29]) in which specimens of the tested bioplastic material are composed with biowaste for 12 weeks. Following this period, the mass of the test material residues must be less than 10% of the original mass;
(3) Effect on the biological treatment process: There should be no negative effects of the packaging materials on the composting process;
(4) Effect on the quality of the resulting compost: The increased packing material should not degrade the final product’s quality. The standard requires that this be verified using ecotoxicity tests: This entails conducting a study to determine whether composted packaging has a harmful effect on plant germination and biomass output. Moreover, the standard sets limits for volatile matter, heavy metals, and fluorine contents of the final compost.

Composting bioplastics has received much attention recently from scientific studies, particularly with regard to bioplastic carrier bags. Nevertheless, by filling in the knowledge gaps and offering a more thorough grasp of the topic, this work adds fresh perspectives to this field of study. Our research uniquely emphasizes both degradation and disintegration, specifically within the context of industrial-scale composting conditions. While numerous studies have explored the biodegradation of bioplastics, our research topic focuses on the intricacies of both degradation and disintegration processes under the particular conditions seen in large-scale composting operations. The complexities and variables inherent in industrial-scale composting, including elevated temperatures, varying aeration levels, organic matter (OM) content, moisture content (MC), ratio of (C/N) in the organic materials, the dynamic interplay of heterogenous organic waste materials, and the diverse microbial interactions, are often overlooked in laboratory-scale studies. By positioning our research within this industrial framework, our primary objective is to generate findings that more accurately mirror real-world scenarios. This aims to enhance the applicability and relevance of our results in the management of bioplastic waste.

Our paper assesses the biodegradation and disintegration of bioplastic in a significant amount of carrier bag waste, marking a departure from the prevailing focus on small quantities in the existing literature. The prevalent limitation of composting smaller quantities of plastics poses a potential impediment to the effective integration of bioplastics into the compost matrix. By concentrating on larger quantities in our research, we aim to enhance the understanding of bioplastic behavior within more realistic composting environments. Furthermore, our study examines, in agreement with the corresponding standards, fragments both greater and less than 2 mm in size, contributing to a comprehensive analysis of bioplastic dynamics during the composting process.

Moreover, one of the main objectives of this paper is to investigate the impact of various abiotic process conditions on the components of PBAT-based bioplastic film through the use of analytical techniques Fourier transform infrared spectroscopy (FTIR), gas chromatography coupled with mass spectrometry (GC-MS), scanning electron microscopy (SEM), and ecotoxicity tests facilitating a comprehensive analysis of the composting process and guaranteeing a thorough exploration of potential environmental implications, particularly in relation to microplastics. FTIR and GC-MS methods were employed to investigate the composting progression of PBAT-based bioplastic bags. The goal was to identify decomposition products and monitor their concentration variations throughout the composting process. This also involves the detection of possible marker component(s), which are suitable to indicate that the decomposition went into completeness.

## 2. Materials and Methods

### 2.1. Tested Material

The test materials used in this study were PBAT-based bioplastic carrier bags available in an international supermarket network in Hungary and certified as compostable under industrial composting conditions according to OK compost^®^ Vincotte by TÜV Austria (Brunn am Gebirge, Austria). Based on the work of Borchani et al. [40], the given PBAT-based biopolymer consists of 20% starch, 10% additives, and 70% PBAT. The studied biopolymer films’ width was 143 μm with haze optical properties of 94% according to ASTM D1003 [41]. The characteristics of the studied biopolymer according to the available material datasheet [42] are presented in Table 2.

### 2.2. Composting Experiment and Sampling

**Figure 1:**
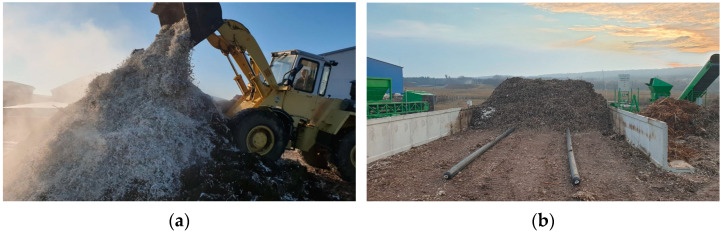

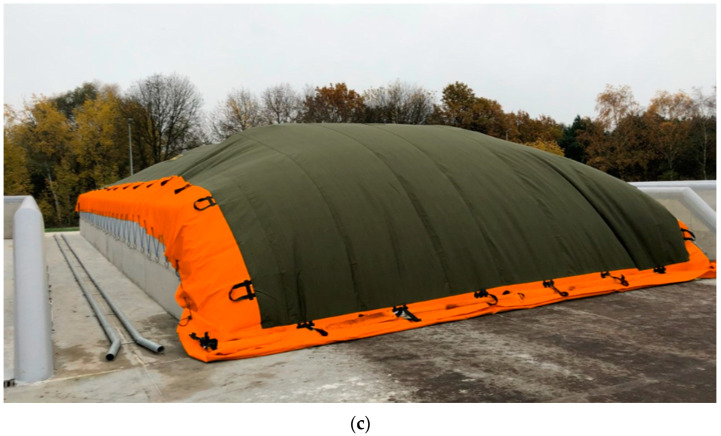
Composting of polybutylene adipate terephthalate-based bioplastic carrier bags, composed of 20% starch, 10% additives, and 70% polybutylene adipate terephthalate at the industrial scale (50 tons), in a membrane-covered side-walled aerated static pile (ASP) system covered with expanded polytetrafluoroethylene membrane cover ProfiCover^®^, Profikomp Environmental Technologies Inc., Gödöllő, Hungary), located near Orosháza, Békés County, Hungary. (**a**,**b**) Composting setup before the experiment. (**c**) ASP during composting.

The composting experiment was carried out in an aerated static pile (ASP) located near Orosháza (Békés County, Hungary). The membrane-covered, side-walled ASP system was an industrial-scale composting unit with dimensions of 25 m in length and 8 m in width (Figure 1). The initial raw materials mixture was composed of approximately 37.5 tons of cattle manure (used as a nitrogen source to adjust the C/N ratio) and shredded wood (used as a bulking agent) and 50 tons of biopolymer carrier bags. Fresh cattle manure with minor straw content was obtained from a local farm in Orosháza city with a production capacity of 5000 tons per year. At the beginning of the experiment, the biopolymer carrier bags were shredded into 10 mm particles and mixed with the wood and manure raw materials. The composting technology used during this study was an aerated static pile composting system with a semipermeable membrane cover, the dimensions of which were 8 m by 4 m. The active aeration system supplied the oxygen needed for the activity of microorganisms during the biodegradation process and was delivered by perforated pipes at the bottom of the compost pile. The aeration system was continuously controlled by the feedback information about the temperature and oxygen levels of the compost pile. The membrane cover allows gas exchange while it retains unpleasant odors, moisture, and heat. The temperature variation during the 12-week composting experiment was measured with a SCADA temperature probe with a length of 1000 mm and equipped with a pt100 electrode (Profikomp Inc., Gödöllő, Hungary).

As for the large amount (50 tons) of the waste biopolymer material and its ratio to the cattle manure applied (1.33), the objective of our study was to assess the biodegradation and disintegration of bioplastic in a substantial, industrial-scale amount of PBAT waste material marking a departure from the prevailing focus on small quantities in the existing scientific literature. The prevalent restraint of composting smaller quantities of plastics poses a potential impediment to the effective integration of bioplastics into the compost matrix. The limitations of the usual practice of composting biopolymers in small batches or including a minor ratio (in most cases less than 1%) in the composting mixture contribute to the misunderstanding of waste treatment principles, as the dilution, which deliberately changes the characteristics of waste without improving its environmental performance, should not be allowed. In our experiment, we explored the potential upper limit of the economical and professional optimum of composting biopolymers as regular composting facilities usually do not accept biopolymer waste due to the required permissions, low economic profitability, and impact on regular operation, putting this type of waste utilization at risk. Moreover, we attempted to apply as little manure as possible but still remain in the workable range of C/N ratios (25 to 35) [46,47,48]. By concentrating on larger quantities in our research, we aim to enhance the understanding of bioplastic behavior within more realistic composting environments.

Sampling was carried out in triplicates on a biweekly basis from the beginning of the experiment until the 12th week. Samples were collected from 1.0 ± 0.2 m depths inside the pile in the middle section to avoid the surface effect. Each time, sample volumes of 15–30 L per point were collected and homogenized to obtain an average sample of 15–20 L by sample reduction. The composite samples from replicates were labeled with sequential even numbers from 0 to 12, representing the number of the composting week. The finished compost was sieved through a 10 mm sieve and placed for maturation for 1 year. Sieving was carried out carefully by hand to avoid, as much as possible, systematic errors due to induced mechanical fragmentation. Physical breakdown was assessed by optical microscopy (see Section 3.6.1). A composite sample was taken from the 1-year matured compost and named M1.

As the original PBAT-based waste was shredded before substrate mixing for composting, the process resulted in 10 mm wide plastic strips with varying lengths. Due to the nature of the PBAT-starch-based waste material, in the first few weeks, the thermophilic conditions made the substance soften up and curl/aggregate with the bulking agent and manure constituents. Subsequently, it hardened up and became crumbling, resulting in disintegration, as stated later (see Section 3.3 and Section 3.4). Because of the initial shredding and high (yet not full) level of disintegration determined at the end of the 12th week, the oversize fraction did not contain a significant amount of polymer after sieving (only the bulking agent) and was excluded from analysis due to the fact that is was aimed to be used in the next batch of composting mixture.

### 2.3. Physicochemical Properties of the Input Material and Monitoring During the Composting Process

Various composting parameters of the input mixture were measured at the beginning, throughout, and at the end of the composting process. Moreover, compost quality parameters were measured after the 1-year maturation period. The values provided in the standard EN 14045:2003 [29] were used as references. The parameters measured and the methods used for the analysis are summarized in Table 3.

### 2.4. Disintegration Test

To evaluate the disintegration behavior of the studied biopolymers throughout the composting process, the standard ISO 16929 [32] was used as a reference, and the sieving analysis was carried out on the first day, 2nd, 4th, 6th, 8th, 10th, and 12th week of the composting process. Each sample was sieved through a 10 mm sieve and examined for big lumps of compost containing fragments of the test material, which were broken up into crumbly particles. The sieved material was further separated through a 2 mm sieve. Test material particles were then collected from the obtained 2 mm to 10 mm fractions, cleaned, dried at 40 °C until constant mass was reached, and finally weighed.

The disintegration rate of each sample throughout the composting process was evaluated based on the respective total dry solids by comparing the mass of the selected test materials from the >2 mm sieved fractions to the mass of the initial test material input (Equation (1)) [32]:

(1) $$D_{i}=\frac{m_{1}-m_{2}}{m_{1}}\times 100\%$$

where *Di* is the degree of disintegration of the test material (in %), *m*_1_ is the mass of total dry solids in the test material input (in g), and *m*_2_ is the mass of the total dry solids in the retrieved test material (in g).

### 2.5. Morphological Changes

#### 2.5.1. Sample Preparation

The disintegration test conducted on a biweekly basis generated two biopolymer compost fractions through the 2 mm sieve by the end of each test: a fraction > 2 mm and a fraction < 2 mm. Plastic fragments from the >2 mm biopolymer compost fraction were recovered, dried, and carefully toothbrushed to remove all deposits formed on their surfaces. The <2 mm fraction was separated into 4 different fractions by sieving it through 1 mm, 500 µm, and 106 µm standard sieves. Microplastics over 500 µm were collected with tweezers as it was possible to perform the process with the naked eye. They were then dried and very carefully toothbrushed.

#### 2.5.2. Optical Microscopy

As it was not possible to investigate with a naked eye the compost matrix combination under 500 µm, it was examined with an AxioCam MRc 5 optical microscope (Carl Zeiss Microimaging Inc., Göttingen, Germany). Pictures were taken with the built-in digital camera (Carl Zeiss Microimaging Inc., Göttingen, Germany).

#### 2.5.3. Scanning Electron Microscopy (SEM)

Examining the composting stages of PBAT-based bioplastic carrier bags on a microscopic level is crucial for comprehending the degradation process and assessing disintegration efficiency. Images of intact and degraded bioplastic fragments from the >2 mm biopolymer compost fraction and the microplastics recovered from the <2 mm fraction (see Section 2.5.1) were collected with HITACHI S-4700 field emission-scanning electron microscope (Hitachi High-Tech Corp., Tokyo, Japan). The biopolymer fragments were metalized with a 10 nm coating of gold before microscopy.

### 2.6. Fourier Transform Infrared (FTIR) Spectroscopy

To investigate the chemical composition changes of the biopolymer bags throughout the composting process, spectra of the intact and degraded fragments from the >2 mm biopolymer compost fraction and the microplastics recovered from the <2 mm fraction (see Section 2.5.1) were collected in total reflectance mode (ATR) with Jasco FT/IR-4200, equipped with a diamond prism and an ATR Pro Penta device (Jasco Corp.; Easton, Saint Louis, MO, USA).

### 2.7. Gas Chromatography Coupled with Mass Spectrometry (GC-MS)

Samples taken after 2, 6, and 12 weeks of composting, as well as 1-year matured compost, have been subjected to GC-MS. The comparative analysis between the larger (>2 mm) and smaller (<2 mm) fractions was emphasized to understand the extent and nature of biodegradation and disintegration.

Sample preparation prior to GC-MS measurements started with the extraction of 1 g sample by using 10 mL of acetonitrile. Samples were subjected to ultrasound agitation for 15 min in a Falcon tube. One mL of the extract was then filtered by a syringe filter (0.45 µm), and the solvent was evaporated by nitrogen stream. A total of 50 µL of silylating agent (*N*,*O*-bis(trimethylsilyl)trifluoroacetamide, BSTFA) was added to the dried residues and derivatized for 30 min at room temperature under moisture-free conditions. Finally, 10 µL of internal standard, containing eicosan (C20, ISTD) at the level of 1 mg mL^−1^, was added to the reaction mixture and diluted to 1 mL with hexane. After homogenization by vortex for 30 sec, 3 µL of the sample was injected. Adipic acid (AA), 1,4-butanediol (BDO), and phthalic acid (PTA) standard solutions were derivatized using the same procedure to establish the calibration curves. Levels for external calibration were 0.5, 1.0, 2.5, 5.0, and 25 μg mL^−1^, which are equal to the target component content of 5 to 250 μg g^−1^. Estimated amounts of the other three components containing either AA or PTA and additional butoxy unit(s) (AA+, AA++, and PTA+) based on the assumption that their detector responses are similar to the corresponding monomer acids (AA and PTA). Validity could not be checked as the standards were not available for these components, but changes in signal intensities obviously show the trends related to their quantity.

Determination of compounds was performed on a Chromass (YL Instruments, Anyang, Republic of Korea) instrument. The initial temperature was 80 °C, held for 4 min, then increased by 15 °C min^−1^ to 280 °C, held for 3 min. The injection port was kept at 250 °C, and the transfer line at 280 °C. The solvent delay was 5.5 min; thereafter, fragments in the range between 50 and 400 *m*/*z* were detected. Components were separated on a TG-5 ms column with the dimensions of 0.25 mm × 0.25 µm × 30 m (ThermoScientific, Waltham, MA, USA).

### 2.8. Ecotoxicity Tests

To determine the effect of the biopolymer compost on the germination and growth of plants, two plant bioassays were implemented: (1) EN 16086-1 [52]: soil improvers and growing media. Determination of plant response pot growth test with Chinese cabbage (*Brassica napa* ssp. *pekinensis*) and spring barley (*Hordeum vulgare*); and (2) the Hungarian standard MSZ 08-0012-4 79 [58], Germination dynamics white mustard (*Sinapis alba*). The 5-day stage is the first validation point of the test, as over 85% of the seeds germinated as needed. The endpoint is phenological stage-related, depending on the control, meaning a differing end point of the tests, 16 days for spring barley and 37 for Chinese cabbage, respectively.

Germination inhibition (*Gel*) is expressed as a percentage of the average germination in the control samples according to Equation (2) EN 16086 [52]:

(2) $$Gel\;\left(\%\right)=\frac{{AGR}_{contol}-{AGR}_{sample}}{{AGR}_{control}}\times 100\%$$

where *AGR* is the average germination rate calculated as the average of the germinated seeds in the different pots after five days from the beginning of the experiment.

## 3. Results and Discussion

### 3.1. Carbon-to-Nitrogen Ratio

The C/N ratio, widely accepted as the most prognostic descriptor of the composts for their N mineralization capacity [59], was measured for the two main components: cattle manure with very low straw content (10:1) and the biopolymer sample (504:1). After correction with the weight and moisture content of the materials a C/N ratio of 35.6 ± 5.3 was detected for the mixture, falling into the range of optimum 25–35 C/N ratio of the initial mixtures [46,47,48]. The carrier bag waste was treated similarly to paper waste when calculating the mixture using a standard operational protocol. The exact aim was to try out the possible limitations of bioplastic to the composting process. The supplier of the carrier bag waste was interested in the possibility of creating a dedicated composting plant for this type of waste.

### 3.2. Temperature

**Figure 2:**
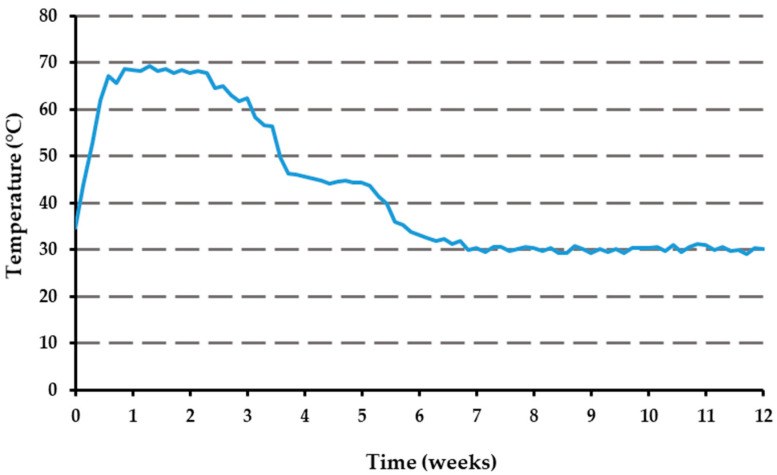
Temperature curve during the 12 weeks of the composting process.

The temperature was recorded daily throughout the entire composting experiment (13 weeks), and Figure 2 illustrates the temperature variation curve over time. The temperature curve exhibits an initial temperature of 52 °C. Subsequently, the composting process transitioned directly into a thermophilic phase, characterized by a sustained temperature of greater than 50 °C. The temperatures recorded were similar to the ones observed by Itävaara et al. (1997) [60], who also reported temperatures exceeding 50 °C and rising as high as 80 °C. Another study by Kianirad et al. (2009) [61] also noted that higher temperatures, typically exceeding 55 °C, are favorable for the efficient biodegradation of PBAT, and even longer aromatic oligomers can degrade during composting at such elevated temperatures [62]. Secondly, the compost maintained a pH between 6 and 7, and an increased pH of 8.16 was observed by the end of the study. A high pH of 8 was also observed by Itävaara et al. (1997) [60] at the end of the composting experiment. The observed shift to an alkaline level likely results from ammonia release, a phenomenon tied to the decomposition of nitrogen-rich compounds. The mature compost’s electrical conductivity, measured at 1251 μs cm^−1^ at 22.8 °C, and its salt content of 0.6% highlight the significance of salinity in the composting pile. Tester (1990) [63] underscored that elevated salt levels, particularly sodium chloride, can hamper microbial activity, thereby decelerating decomposition. The salt metrics from our compost suggest a balance that does not hinder its potential utility, yet careful monitoring remains essential.

### 3.3. Respiration Intensity

**Figure 3:**
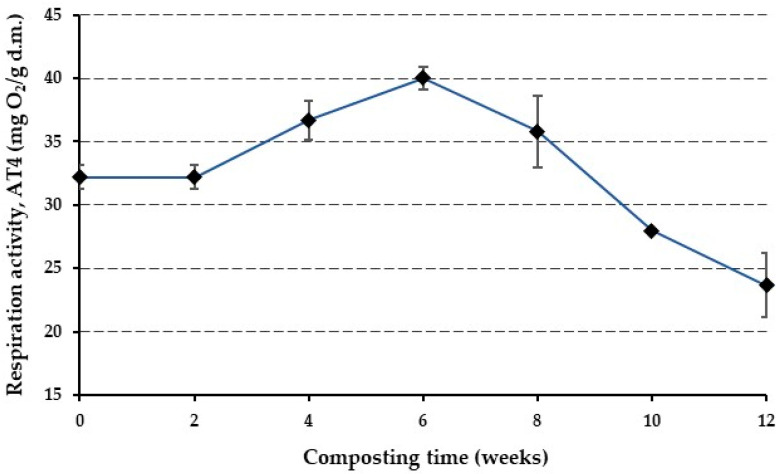
Evolution of respiration activity (AT4) during the 12 weeks of the composting process.

The oxygen consumption rate was monitored throughout the 12-week composting process to examine the aerobic activity and the rate of decomposition of the biodegradable organic components within the composting material. The oxygen uptake rate is a vital indicator, as it is directly linked to the mass of the degradable substance through a reaction coefficient (*k*) known, from which the respiration activity, AT4, is derived by first-order reaction kinetics. This coefficient is influenced by various environmental factors, including temperature, MC, biodegradability, fatty acid synthesis, pH, and the initial C/N ratio of the material [64]. Figure 3 illustrates the evolution of the oxygen uptake rate over the composting period. The graph revealed distinct microbial activity patterns during the composting of the bioplastics.

The initial two weeks were characterized by a lag phase, with a constant respiration intensity of approximately 32 mg O_2_ g^−1^ dry matter (d.m.). This phase signifies the acclimatization and adaptation period of the microbial community to the composting environment. After this phase, there was a noticeable increase in microbial activity. The oxygen consumption rate gradually escalated, reaching its peak at about 40 mg O_2_ g^−1^ d.m. during the sixth week. After reaching the peak, the respiration intensity began to gradually decrease. This decline continued until the end of the composting process, where the oxygen consumption rate was finally measured at 23 mg O_2_ g^−1^ d.m. This reduction in respiration intensity signifies the depletion of easily degradable materials and the stabilization of the compost. The oxygen consumption rate is closely linked to the CO_2_ production rate, as microbes utilize oxygen to metabolize organic materials, emitting CO_2_ as a waste product. This balance between oxygen consumption and CO_2_ production is indicative of the biodegradability of the material [65]. The results are comparable with the findings of [66], where they reported high degradation rates for specific plastic polymers, even surpassing the biodegradation rate of the cellulose standard. This rapid biodegradation, evident in the early stages of their trials, resulted in over 80% biodegradation in just about 20 days for certain plastics [66]. The results are also comparable to the study by Tabasi and Ajji (2015) [67], where the composting of PBAT also exhibited a lag phase followed by a peak in oxygen consumption.

### 3.4. Organic Matter Mineralization

The initial C/N ratio as the basis of mineralization was slightly higher than the literature recommendations and gradually decreased to 15.9 ± 0.3 during the composting process. The mineralization and degradation rate of organic matter (OM) are pivotal indicators of compost quality and stability. At the commencement of the composting experiment, the OM content was recorded at 77.43% ± 9.47. Throughout the 12-week period, a consistent reduction in this value was observed, culminating in an OM content of 62.43% ± 1.25 by the end of the process. This signifies a relative loss of 17.39% ± 10.72 in OM.

The concluding OM content denotes a high-caliber compost product, signifying proficient decomposition and stabilization of OM. The values for OM content during the experiment are provided in Table 4. The results observed were compatible with the findings by Ruggero et al. (2021) [68], who employed the Walkley–Black method for the measurement of total organic carbon, providing a benchmark for OM content.

The composting process showed a consistent decline in MC over its duration. The initial MC was measured at 46.08% ± 1.29. By the 12th week, this value had decreased to 35.90% ± 1.30. A significant positive correlation between microbial activity and MC was observed, with a correlation coefficient of R^2^ = 0.75 (*p* < 0.05). This result emphasizes the direct relationship between moisture and microbial activity, suggesting that the MC plays a pivotal role in the microbial degradation process during composting. Drawing from Ruggero et al. (2020) [69], the biodegradation of the PBAT-based biopolymer is highly influenced by moisture. Under conditions with MC between 55% and 45%, PBAT showed degradation rates of up to 90.4%. When MC drops below optimal values, there is a noticeable slowdown in biological activity, which can also be reflected in the degree of biodegradation. The obtained values were less than the values mentioned in the standard. The mature compost had a low moisture content of 5.46 ± 1.21 due to the long drying period. The OM and MC values during and at the end of the composting are provided in Table 4.

### 3.5. Disintegration Test

**Figure 4:**
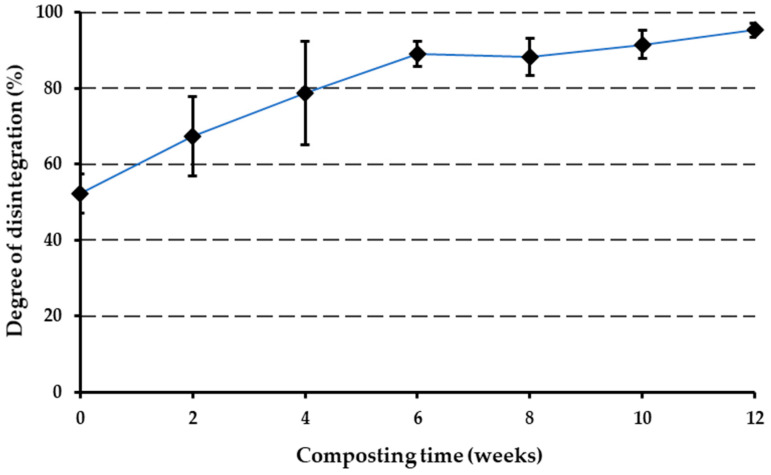
Degree of disintegration of biopolymer during the 12 weeks of the composting process.

**Figure 5:**
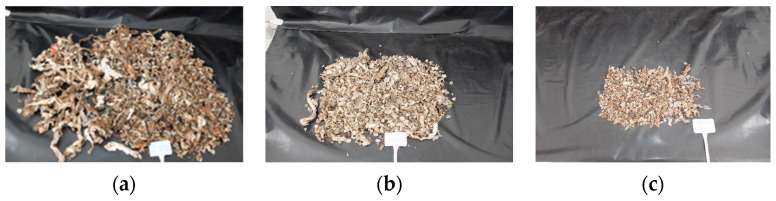
Representative pictures taken during the disintegration of the biopolymer fraction greater than 2 mm (**a**) on the first day, (**b**) on the 6th week, and (**c**) on the 12th week of the composting process.

The degree of disintegration of the PBAT-based biopolymer during the 12-week composting process was calculated using Equation (1), and the values are presented in Figure 4. A substantial increase in the degree of disintegration, particularly noticeable from the fourth week onwards, was observed. By the 12th week, the degree of disintegration reached 95% ± 1.86. A notable bioplastic disintegration of 67.57% ± 10.21 was observed after the second week of the process and reached 78.82 ± 13.49 by the fourth week. Figure 5 illustrates the diminished presence of plastic fragments larger than 2 mm at this stage. Figure 5c represents the disintegrated fragments at the end of the composting experiment. The study by Ruggero et al. (2020) [69] also found that bioplastics underwent disintegration of more than 90% by the end of composting.

**Figure 6:**
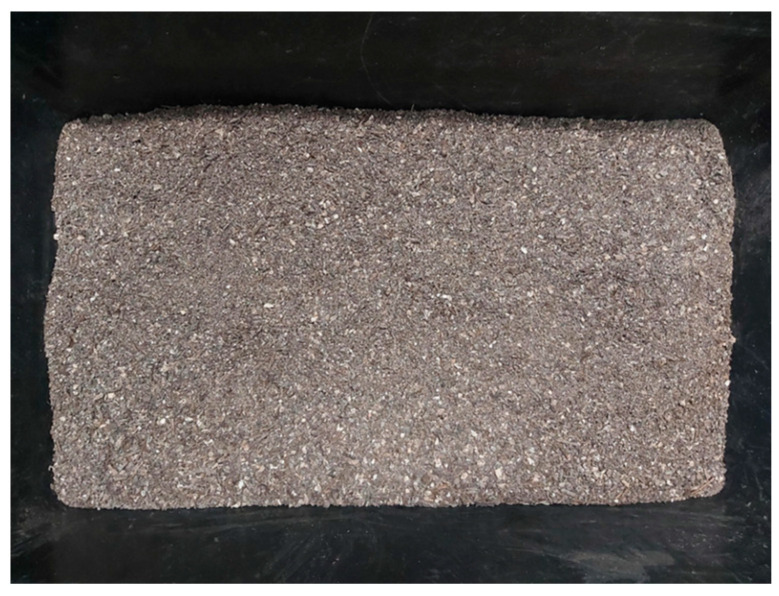
Visual compost matrix after 1 year of maturation.

A visual examination of the sieved compost revealed discernible contaminants, significantly affecting the overall coloration and rendering it a grayish hue (Figure 6). To further investigate, an additional image, Figure 6, was captured after a maturation period of one year, aiming to assess any visible changes in the compost’s appearance. Evidently, the grayish aspect persisted, visually resembling microplastic fragments.

### 3.6. Morphological Changes

#### 3.6.1. Optical Microscopy

**Figure 7:**
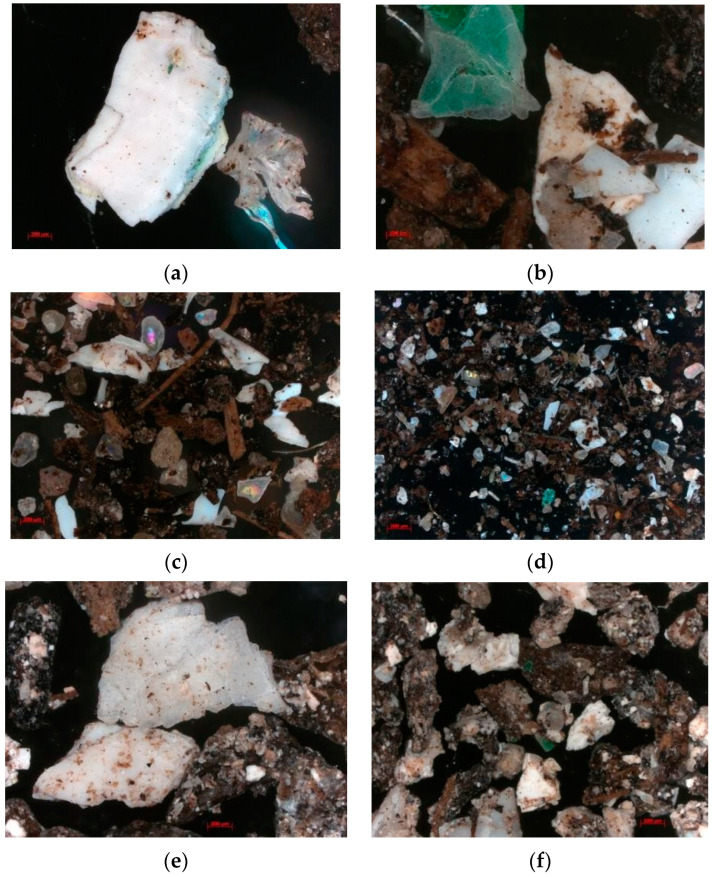

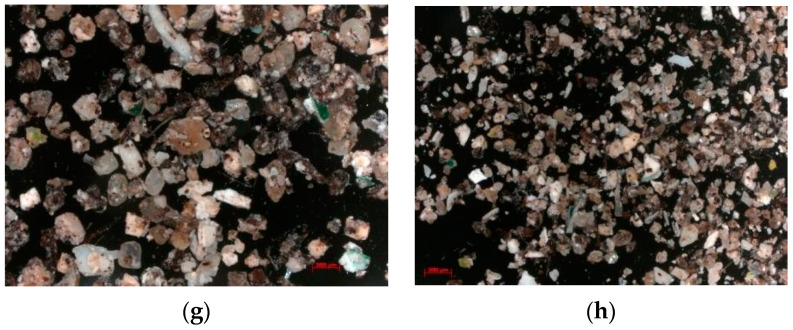
(**a**–**d**) Images of fragments retained in the compost matrix after the 2nd week of composting; (**e**–**h**) images of fragments retained in the compost matrix after the 12th week of composting.

Mechanical fragmentation from the sample preparation process could be distinguished from biological disintegration by examining the edges of particles under a microscope. Microscopic evaluation indicated that the effect of sieving was practically negligible yet an unavoidable consequence of sample preparation. Plastic fragments smaller than 2 mm were difficult to detect and extract from the compost matrix. Figure 7 provides images of various compost matrix fractions captured during the second and twelfth weeks using an optical microscope at different magnifications. Due to the matrix’s intricate composition, the microscopic identification of plastic materials was challenging. That is why SEM was used to further detect the plastic fragments and their degradation over time. Identification was primarily based on shape and color attributes, as highlighted by Jung et al. (2021) [70]. As evident in Figure 7a–d, the compost matrix contained particles in shades of white, cream-white, green, and red/pink. These particles often displayed fragmented or flake-like appearances with uneven edges. Additionally, transparent particles, potentially microplastics, were present but posed identification challenges.

The potential presence of unknown additives in the bioplastic matrix further complicated the identification process, emphasizing the limitations of optical microscopy for recognizing biodegradable microplastic fragments. Figure 7e–h represent the compost matrix after 12 weeks. The finished compost appeared to contain fewer large fragments (>500 μm) but increased smaller fragments in fractions < 250 μm. Additionally, the images revealed the formation of a more complex matrix of OM and mineral constituents. This matrix was characterized by the absorption of biodegradable microplastics into other organic substances originating from the manure. As dispersity increases (decreases in particle radii), the active surface area typically increases, potentially leading to enhanced adsorption capacity for nanoplastics, as suggested by Fojt et al. (2020, 2022) [71,72]. These interactions could hinder the microbial degradation of bioplastics. Thus, understanding the formation of a complex matrix during composting and the dynamics of microplastic adsorption is crucial for effectively mitigating microplastic pollution from biodegradable polymers.

Moreover, the reliance on attributes such as shape and color for identification well corresponds to findings presented by Ruggero et al. (2020) [69], where visual inspections indicated marked changes in the bioplastic materials just after 5 days of exposure to organic waste. In their study, the bioplastic pieces showed significant OM deposition, emphasizing the interactions between bioplastics and surrounding OM, which may facilitate microbial exchanges.

#### 3.6.2. Scanning Electron Microscopy (SEM)

**Figure 8:**
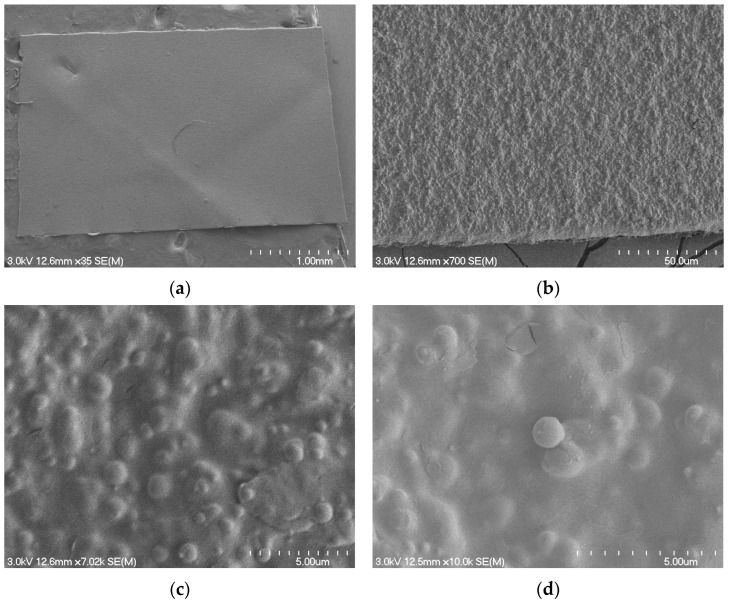
Scanning electron microscope micrographs of a sample material captured at varying magnifications. (**a**) 35× magnification offering a broad overview of the surface. (**b**) 700× magnification showcasing a unique fibrous texture. (**c**) 7.02 k magnification detailing intricate surface nodules and irregularities. (**d**) 10.0 k magnification showing granular formations and the intricate nature of the surface.

The SEM micrographs presented in Figure 8, captured at varying magnifications, display the PBAT-based bioplastic bag’s intricate microstructure. Specifically, these images highlight the presence of spherical spots of a few hundred nanometers. These spherical inclusions are identified as the starch fraction of the blend mix, dispersed within a continuous PBAT matrix [73]. The size of spherical spots depends on the exact composition of the biopolymer, and the presence of additives (e.g., compatibilizers) affects the microstructure as well as the degradation rate [74].

**Figure 9:**
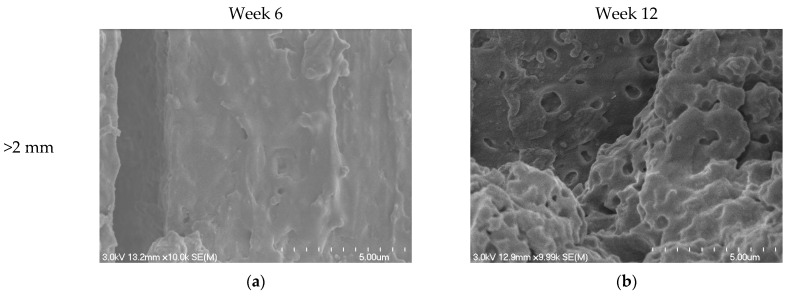

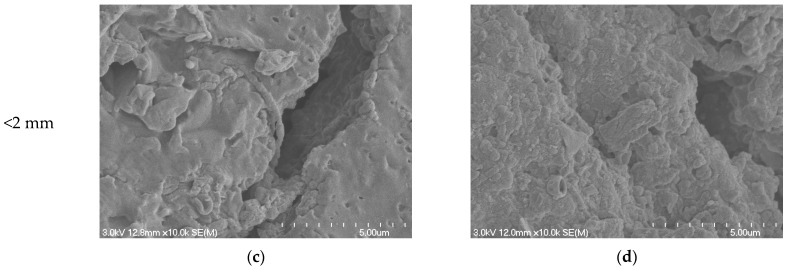
Scanning electron microscope micrographs depicting morphological changes in carrier bag fragments over the course of composting. (**a**) Week 6 fragment from the >2 mm compost fraction, revealing initial surface disruptions and cracks. (**b**) Week 12 fragment from the >2 mm compost fraction, highlighting intensified degradation with pronounced irregularities. (**c**) Week 6 fragment from the <2 mm compost fraction, showcasing initial signs of wear and surface cracks. (**d**) Week 12 fragment from the <2 mm compost fraction, exhibiting marked surface degradation and increased cracking patterns.

Figure 9 shows the morphological alterations observed in carrier bag fragments, sourced from both >2 mm and <2 mm compost fractions, at the 6th and 12th weeks of the composting process. By the sixth week, the onset of surface disruptions, evidenced by cracks, becomes noticeable on the formerly smooth biopolymer bag’s surface (Figure 9a,c). This degradation escalates by the 12th week, manifesting as heightened surface irregularities and more pronounced cracking (Figure 9b,d). As the biodegradation process continues, the microstructure transforms into a 3D porous network. Notably, the characteristic starch-based spherical spots diminish and are replaced by nanoscale voids, typically in the magnitude of hundreds of nanometers. This morphological evolution suggests that the starch component of the blend underwent substantial biodegradation during the composting process. The increased surface abrasiveness, coupled with the emergence of fissures and voids, potentially augments sorption sites. This could enhance microplastics’ propensity to adsorb specific contaminants or organic molecules [75].

The SEM micrographs of the PBAT-based bioplastic material in the current study, which depicted a heterogeneous microstructure with the presence of circular spots of a few hundred nanometers, align with the observations made by Ruggero et al. (2020) [69]. These spots, attributed to starch constituents, were dispersed within a 3D polymeric matrix, believed to be primarily comprised of PBAT. Such a microstructural depiction resonates with the portrayal of a PBAT-based bioplastic by Ruggero et al. (2020) [69], wherein the circular starch spots underwent progressive disappearance during the composting process. This was accompanied by the emergence of nanoscale holes at the sites of these starch granules, suggesting the composting-induced degradation of starch. Furthermore, Ruggero et al. (2021) [76] and Myalenko and Fedorova (2023) [77] embarked on a comprehensive exploration of bioplastic degradation during composting. Their SEM evaluations corroborate the morphological transitions observed in the current study, reflecting the dynamic microstructural alterations that bioplastics undergo throughout composting. This includes changes in surface roughness, the emergence of cracks, and the development of 3D porous networks.

### 3.7. Fourier Transform Infrared (FTIR) Spectroscopy

**Figure 10:**
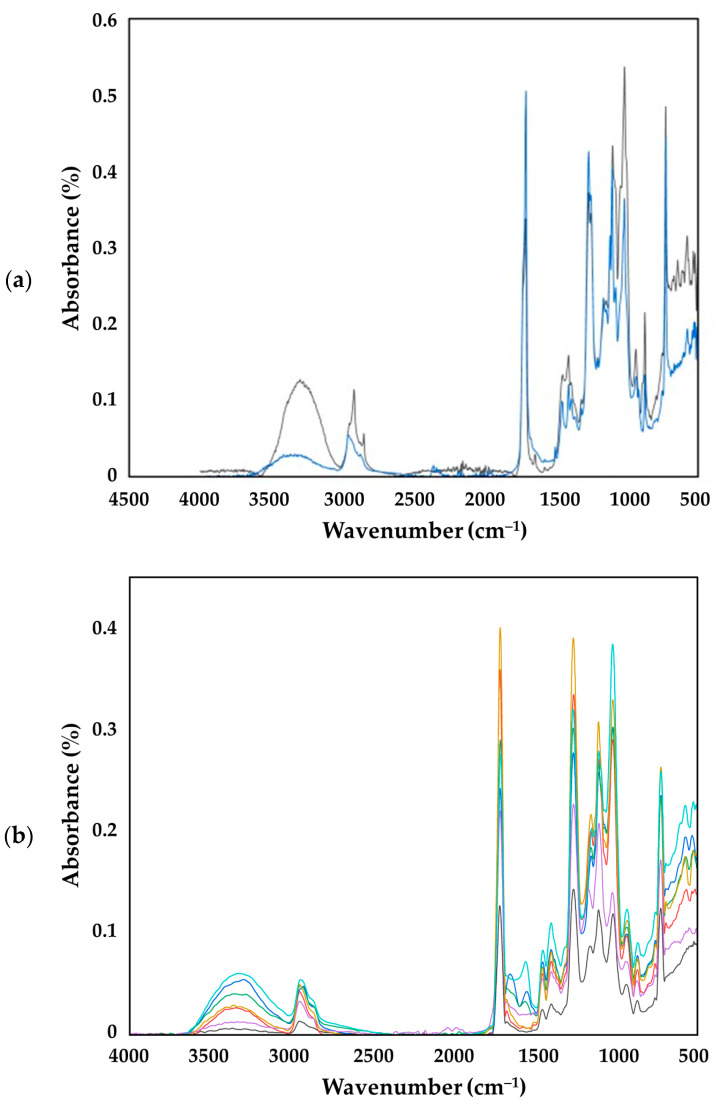

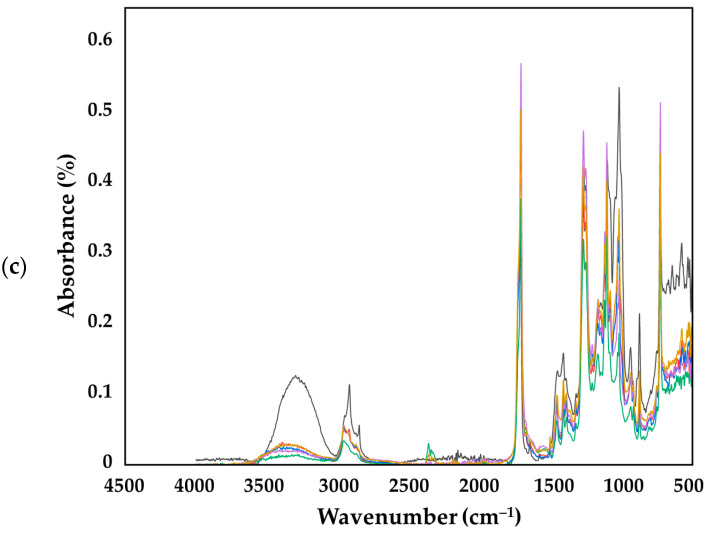
Fourier transform infrared (FTIR) spectra of the polybutylene adipate terephthalate-based bioplastic bag material. (**a**) Comparing the raw sample (gray line) to the sample after 12 weeks (blue line) of composting. (**b**) The bioplastic bag fragments (>2 mm) comparing the raw sample fraction (gray line) to sample fractions after 2 weeks (red line), 4 weeks (blue line), 6 weeks (green line), 8 weeks (purple line), 10 weeks (yellow line), and 12 weeks (turquois line) of composting. (**c**) The PBAT-based bioplastic carrier bag fragments (>2 mm) comparing the raw sample fraction (gray line) to sample fractions after 4 weeks (red line), 6 weeks (blue line), 8 weeks (green line), 10 weeks (purple line), and 12 weeks (yellow line) of composting.

To investigate the chemical composition changes of the biopolymer material during composting and the impact of shredding on their molecular structure, raw PBAT-based bioplastic bag (Figure 10a), as well as fragments from both the >2 mm (Figure 10b) and the <2 mm fractions (Figure 10c), were collected and subjected to FTIR spectrometry analysis in total reflectance mode. The collected fragments were analyzed on a weekly basis, spanning from week 0 to week 12 of the composting process. Figure 10a shows the FTIR spectra obtained from the raw biopolymer bag compared to week 12 of composting, and spectra of the biopolymer bag sample for larger (>2 mm) and smaller (<2 mm) fragments from week 0 to week 12 are presented in Figure 10b and Figure 10c, respectively.

FTIR analysis shows structural changes and biodegradation pathways; however, FTIR spectra of decomposition products (AA, PTA, and BDO) overlap with that of the starting material. In addition, starch has, unfortunately, no characteristic peak out of the crowded fingerprint region. The peaks recorded for the composted samples are in accordance with the literature data reported for PBAT (Table 5). Peaks in the region between 3500 and 3000 cm^−1^ (O-H stretching) indicate the presence of starch fraction in the bioplastic bag [78]. The exact frequency is influenced by the adsorbed MC and associated hydrogen bonds. Asymmetric and symmetric C-H stretching appear at 2957 and 2874 cm^−1^, respectively. Strong signals observed at 1720 cm^−1^ and 1274 cm^−1^ represent the carbonyl (C=O) and C–O groups in the ester linkages [40]. According to Ruggero et al. (2020) [69], the shifts in the peak at 1717 cm^−1^ are potentially indicative of degradation within the PBAT fraction of the biopolymer and are potentially related to initial hydrolytic actions or enzymatic activities targeting the ester linkages within PBAT, thereby initiating its degradation. Peaks between 1500 and 1300 cm^−1^ are representative of the aromatic structures within PBAT, but weak deformation vibrations of the CH_2_ group may also contribute to the signal intensities in this region. Peaks at 1110 and 1025 cm^−1^ correspond to C–O stretches in secondary and primary alcohol moieties, but also, in this region, the in-plane C–H bending of the aromatic rings appears. They exhibit alterations in relative intensities as well as disappearances of shoulders that indicate a gradual degradation or structural modification of the starch fraction within the biopolymer matrix. Signals at 940 cm^−1^ correspond probably to the O–H bend, whereas peaks at 875 cm^−1^ are related to the C–H deformation vibration in para-substituted phenyl rings. Similar bands are detected for PTA, and there is also a signal at 934 cm^−1^ in the FTIR spectrum of AA.

The comparison between the raw material and the sample at week 12 (Figure 10a) shows slight shifts and changes in intensity, which implies possible ester bond hydrolysis related to bioplastic biodegradation. The spectra of the PBAT-based biopolymer matrix indicate changes in the region of 1700–1750 cm^−1^ attributed to carbonyl (C=O) stretching vibrations. Similarly, the changes in the aliphatic chains of the polymer are observed at peaks around 2957 cm^−1^ and 2874 cm^−1^ (CH_2_ stretching). The observed differences in these peak intensities between the raw material and the 12-week sample may indicate potential disruptions of the aliphatic backbone, which could be instigated by abiotic factors or microbial activity during the composting process. In addition, the relative intensity of O–H stretching vibration compared to the initial spectrum substantially decreased as well, and peaks at 1110 and 1025 cm^−1^ were lost from their intensities as starch decomposed.

It is observed that the spectrum for fraction >2 mm (Figure 10a) after 4 weeks of degradation is almost identical to the spectrum recorded at 2 weeks of degradation. However, certain changes emerge during the sixth week of the composting process, with a slight decrease in the intensity of the peaks at 1104 cm^−1^ attributed to C–O vibration and at 1268 cm^−1^ attributed to C–O in the ester linkage. This decrease indicates the start of degradation in the fraction above 2 mm, which means that the polymer’s degradation began late at the thermophilic phase.

The FTIR spectra of fragments <2 mm (Figure 10c) show more overall degradation, but the persistence of certain peaks shows that microplastics are still present in the compost matrix. Table 5 presents the measured and reported wavenumbers and the corresponding functional groups discussed in the literature.

Nevertheless, signal intensities in ATR are influenced not only by the absorptivity of functional groups and the concentration of molecules, but other factors (e.g., number of reflections) have an impact. The penetration depth in IR absorbance measurements is only some microns under the material surface; therefore, inhomogeneities of the surface can result in high variability. In addition, the penetration depth is higher for low wavenumbers, which results in differences in relative intensities compared to the transmission spectra. Changes in the 3200–3500 cm^−1^ spectral region, associated with hydroxyl (OH) stretching and water adsorption, might reflect variations in the hydrophilic nature of the material or its interaction dynamics with water molecules during the composting, both of which could be influenced by the degradation processes. The disappearance of peaks or shoulders attributed to starch implies that the fraction has degraded by the end of the composting process. Based on the changes in FTIR, the end of degradation and decomposition rate cannot be determined. As the functional groups in possible metabolites are similar, signals overlap in the FTIR spectrum of the polymer. Moreover, reference materials, as well as detailed knowledge of the decomposition routes, are lacking. All these prevent the quantitative determination of metabolites by FTIR. Determination of metabolites requires the application of other methods capable of separating the components from the complex matrix and providing structural information as well. Therefore, we extended the investigations to determine the key metabolites by using GC-MS.

### 3.8. Gas Chromatography Coupled with Mass Spectrometry (GC-MS)

**Figure 11:**
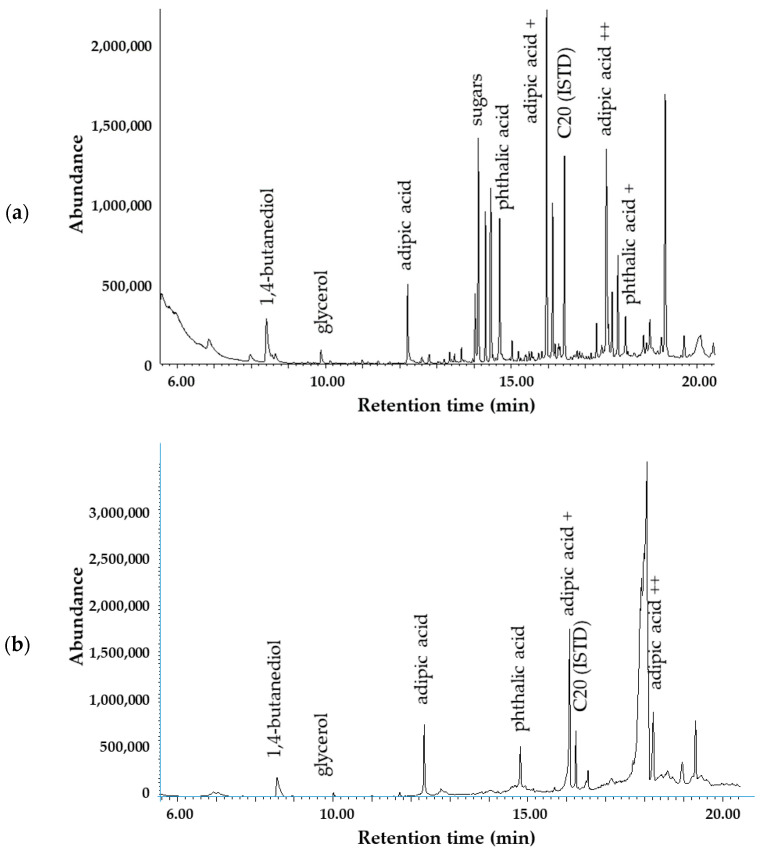
GC-MS chromatogram of the sample showing the distribution and abundance of key compounds after 2 weeks of composting (**a**) and after 1-year maturation (**b**).

**Figure 12:**
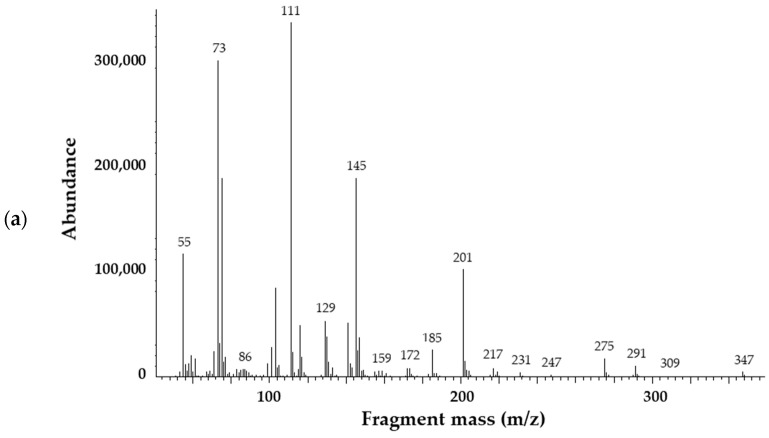

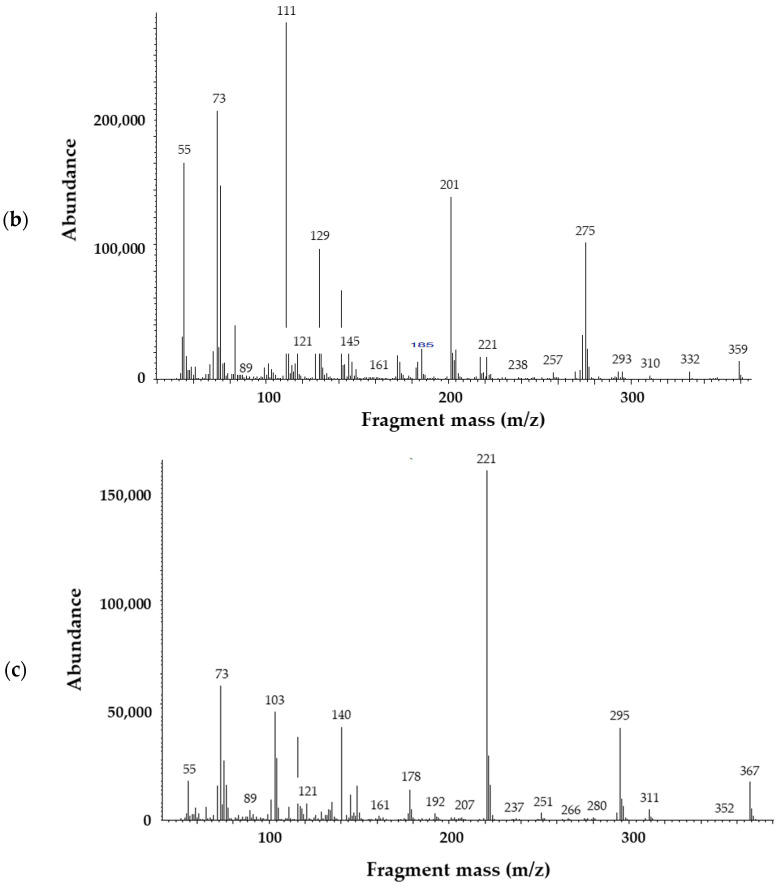
Mass spectra recorded for bis(trimethylsilyl) derivatives of the intermediate metabolites 4-hydroxybutyl)adipate (AA+) (**a**), bis(4-hydroxybutyl)adipate (AA++) (**b**), and (4-hydroxybutyl)terephalate (PTA+) (**c**).

**Figure 13:**
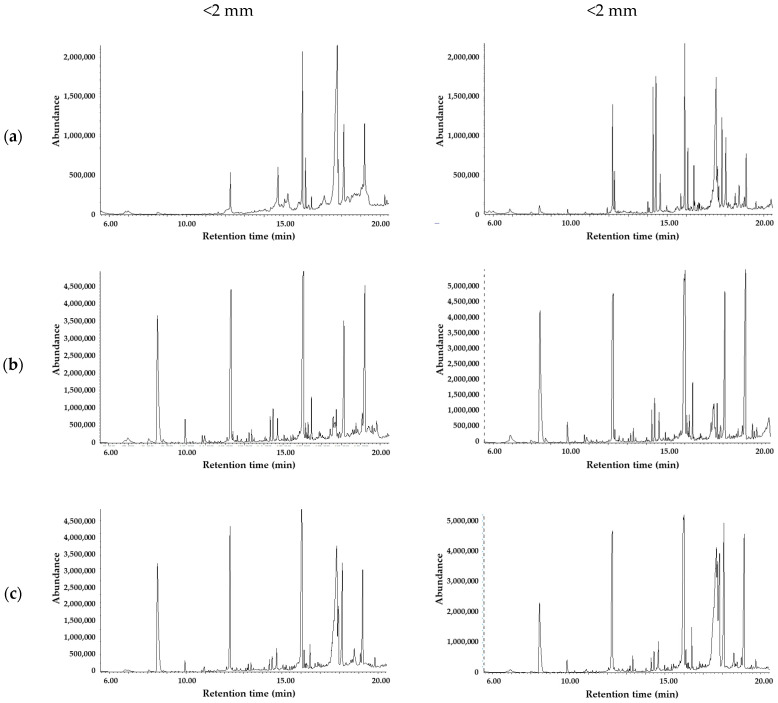
GC-MS chromatograms for <2 mm and >2 mm fractions at 2 (**a**), 6 (**b**), and 12 (**c**) weeks.

**Figure 14:**
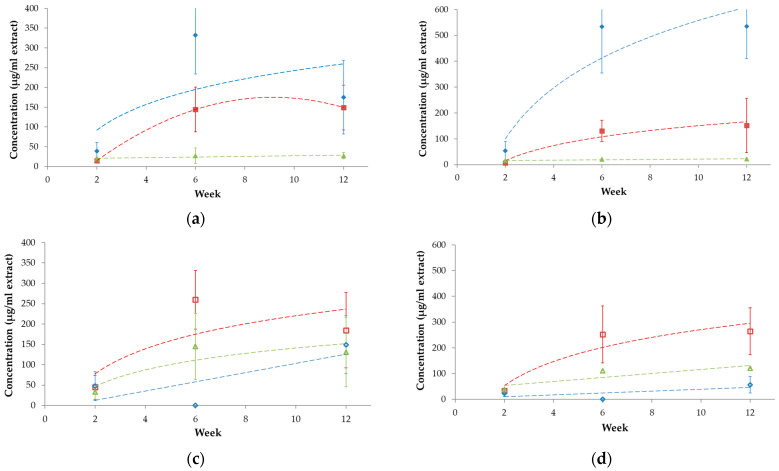
Concentrations in the polybutylene adipate terephthalate-based bioplastic bag fragments from >2 mm (**a**,**b**) and <2 mm (**c**,**d**) compost fraction observed at the 2nd, 6th, and 12th weeks of composting experiment. (**a**,**b**) shows the levels of AA (■ red) and PTA (▲ green). 1,4-butanediol (♦ blue). (**c**,**d**) present the estimated levels for AA+ (□ red), PTA+ (∆ green), and AA++ (◊ blue) based on *m*/*z* = 111.

As information provided by FTIR about the chemical changes of the biopolymer bags during composting was limited, a GC-MS analytical method has also been developed to characterize the decomposition process. The goal was to detect the end of the degradation or to find marker molecules to follow the process by a more specific and selective analytical method. Determination of target components by GC-MS requires derivatization to improve their volatility and detectability. Recently, GC-MS methods applying high temperature (pyrolysis, thermal extraction desorption GC-MS) were published to identify markers of different microplastics in the environmental matrices [81] and to study the thermal decomposition of biodegradable polymers, including PBAT [81,82,83]. Derivatizing agents were rarely utilized in investigations of pyrolytic products. Among others, hexamethyl disilazane (HMDA) proved to be efficient for the chemical modification of alcoholic and carboxylic active hydrogens [83], and earlier, it has been applied to enhance the detection capability of hemp fibers [82]. HMDA is a weak silylating agent. But under pyrolysis conditions, the reaction is fast, as the usual procedure requires 70 °C and longer reaction time. In our study, we chose BSTFA for these purposes because this reagent is more reactive than HMDA. In addition to BSTFA, trimethyl orthoacetate (TMOA) was tested as a derivatizing agent. A neat TMOA reagent was applied at 60 °C for dried residues, but the signal intensities and probably the conversions were not satisfactory. In contrast, silylation resulted in the desired derivatives within 30 min without heating. For the extraction, acetonitrile was as effective as the dichloromethane; thus, it was chosen as a solvent. Typical chromatograms (Figure 11) of the prepared samples contain silyl derivatives of the corresponding monomers (AA, BDO, and PTA). These final metabolites could easily be identified on their mass spectra stored in the NIST database. In addition, three further molecules have been detected, which originate from PBAT polymer and are intermediate metabolites. Two of them have a very similar structure to AA, but mass spectra (Figure 12) contained fragments of higher masses (e.g., 347 or 359) as well, indicating the presence of one (AA+) or two butanediol units (AA++) in the molecule. The third one had similar fragments to the PTA derivative, but the fragmentation pattern regarding the intensities was somewhat different. Fragments of higher masses (e.g., *m*/*z* = 367 [M-15]^+^) also appeared in the mass spectrum, which belongs to the structure (PTA+) consisting of PTA and a butanediol unit as well. As is characteristic for silyl derivatives, molecular ion was not detected, and only the fragments with a methyl group loss appear at low intensities. Samples taken in the first period of composting (2, 6, and 12 weeks) also contained silylated sugars (retention time range, RT = 12–16 min), which may originate from the hydrolysis of starch and the subsequent breakdown of complex carbohydrates into simpler sugars by microbial activity. Silylated glycerol was also detected (RT = 9.88 min), as well as acetyl tributyl citrate in more samples. The observed pattern of decomposition products is substantially different from those obtained under pyrolysis conditions. At 600 °C, the formation of butenyl esters (alpha cleavage and hydrogen transfer) as well as cyclic oligomers are typical, and some of them where proposed as markers to identify polymers in the environmental microplastic [81]. Among the silylated pyrolysate products of PBAT, benzoic acid produced the most intensive peak [83], as decarboxylation of acids also often occurs. Under composting conditions, the original monomers dominate among the decomposition products, and three additional butandiol esters of corresponding acids (AA+, PTA+, and AA++) were also detected (Figure 11, Figure 12, Figure 13 and Figure 14).

There were no significant differences in the chromatograms (Figure 13) as well as in the levels of decomposition products measured for the smaller (<2 mm) or bigger (>2 mm) particles. Intensities were low after 2 weeks, but there was a significant increase by the 6th week in the concentrations of key metabolites (BDO, AA, and PTA) and their derivatives (AA+ and PTA+), which is related to rapid biodegradation during the thermophilic phase. This observation is in accordance with the FTIR results. Subsequently, a slight drop occurred by the 12th week in the concentration of certain degradation products (e.g., BDO), yet their residual concentrations remained relatively high. This suggests that active composting was still taking place, and microbial degradation continually progressed even at this late stage.

Low levels (<28 µg mL^−1^ in the extract equal to 280 µg g^−1^ in the compost) were determined for PTA, and time-dependent changes showed a slightly increasing trend (Figure 14). Intensity for AA increased from low levels (15 and 7 µg mL^−1^) after 2 weeks of composting to 131–144 µg mL^−1^ at the 6th week, but then did not alter significantly until the 12th week. Levels of AA (<152 µg mL^−1^) were generally lower compared to the BDO (<535 µg mL^−1^). The concentration of BDO reached a maximum in the sixth week for a bigger fraction (>2 mm) and seemed to stabilize after the sixth week for a smaller fraction (<2 mm). However, the standard deviations calculated from the measured data for the three replicates are generally high; therefore, the results often do not differ significantly from each other. The observed signal intensities of the other three identified molecules (AA+, AA++, and PTA+), containing the corresponding acid and additional butoxy unit(s), were somewhat lower but still in the same orders of magnitude as for the monomers (AA, BDO, and PTA). AA+ resulted in the highest intensity, followed by PTA+, and amounts of AA++ were sometimes low and overlapped with a broad signal. However, PTA+ was more intensive compared to PTA, and its levels were similar in both size fractions. Its estimated concentration in the extract did not increase after the sixth week but remained in the range between 121 and 145 µg mL^−1^. The same has been observed for A+. For the larger fraction, its level seemed to be lower in the 12th week compared to the 6th week samples, but the difference is not significant because of the high standard deviation values. Due to the interferences, the results for AA++ seem to be not reliable in some cases. Despite the fact that *m*/*z* = 111 was selected for the estimation of AA++ quantity, which was not a characteristic fragment ion in the mass spectrum of the interfering component(s), its effect could not be eliminated in some cases.

Despite the thorough mixing of the composted material, high standard deviation values observed, typically around 50%, indicate that the compost was not so homogeneous. This shows that it is difficult to reach homogeneity in composting performed on the industrial scale. Local conditions, including temperature inhomogeneities in the different parts of compost, result in differences in the decomposition rates; crystallization may also occur, resulting in changes in chemical composition.

Matured 1-year-old compost sample (Figure 11b) did not contain sugars, indicating the successful degradation of starch present in the biopolymer and that of other complex carbohydrates from the manure. Although the sugars were entirely broken down, all other compounds related to the decomposition of PBAT and mentioned above were still detected. However, their intensities were similar to the values determined for second-week samples, typically about one order of magnitude lower (<35 µg mL^−1^) compared to the samples taken after the 6th or 12th week of composting. Concentrations were 19.3, 3.1, and 14.6 µg mL^−1^ for BDO, AA, and PTA, respectively. Estimated values for AA+, PTA+, and AA++ were 27.5, 29.1, and 31.2 µg mL^−1^, which means about 275 to 312 µg g^−1^ in the compost. The presence of the decomposition products indicates that the process did not go into completeness.

Although toxicology data are for the monomers, the main decomposition products are favorable as they are regarded as not hazardous to the aquatic environment (e.g., upon leaching from the compost), but the presence of large amounts of microplastic in the environment is of concern. It is worth noting that microplastics are able to bind pollutants (e.g., PAHs, organochlorine pesticides), which can be transported to surface and groundwater [84]. Based on the material safety data sheets, AA has an acute toxicity value for aquatic invertebrates of 46 mg L^−1^, whereas its chronic LC_50_ value is 17.6 mg L^−1^. The acute and chronic EC_50_ values for aquatic invertebrates determined for PTA are 640 mg L^−1^ and 42 mg L^−1^, respectively. Among these compounds, BDO was found to be the least toxic, with an acute LC_50_ value of 813 mg L^−1^ for *D. magna*.

### 3.9. Ecotoxicity Tests

The ecotoxicity tests were carried out to determine the influence of compost, when mixed at a 50:50 ratio with potting soil, on the germination and growth of three distinct plant species. The results are presented in Table 6. As seen from the instrumental analytical (FTIR, GC-MS) results (see Section 3.7 and Section 3.8), PBAT has not fully degraded during the composting process. The ecotoxicity tests can indicate what effect the remaining PBAT may have on plant germination.

Chinese cabbage (*Brassica rapa* ssp. *pekinensis*) exhibited a more resilient response to the compost mixture. With a mere 7% germination inhibition, it managed to produce a green mass corresponding to 93% of the control’s yield. This suggests that the compost constituents have only a negligible inhibitory effect on the germination and subsequent growth of Chinese cabbage. Consequently, a 50:50 compost-to-soil ratio might be an appropriate medium for fostering the growth of this plant species. However, the assessment for spring barley (*Hordeum vulgare*) highlighted a tangible effect on germination, with the compost causing a germination inhibition of about 33%. This can be deduced from the recorded 67% green mass in the presence of the compost relative to the control sample, which only contained potting soil.

In contrast, the white mustard (*Sinapis alba*) displayed a pronounced sensitivity to the compost mixture, with its germination inhibition of 61%. Such a contrast to the Chinese cabbage implies species-specific responses to the compost mix, possibly due to distinct physiological attributes or growth requirements. Further characterization of the mature compost was performed to understand its phytotoxicity. Its electrical conductivity was recorded at 1251 s cm^−1^ at a temperature of 22.8 °C, and it manifested a relatively low salt content of 0.6%. In plant growth, the concentration of salt can be a pivotal factor, as elevated levels might impede germination. However, in this case, the salt content was deemed non-inhibitory for the studied plants. Another pertinent observation was the respiration intensity of the matured compost, measured at 4 mg O_2_ g^−1^ d.m. This low value is indicative of its stability, suggesting that the microbial activity within the compost has seized. Thus, the results suggested the maturity and the non-phytotoxicity of the compost.

The Chinese cabbage tests, showing only 7% germination inhibition, parallels the study by Vaverková and Adamcová (2015) [85], which observed high germination capacities in plant growth tests for compost. These collective results hint at the potential suitability of certain composts for specific crops, like Chinese cabbage. However, the pronounced sensitivity of white mustard to our compost mix, registering a 61% germination inhibition, underscores the intricacies of plant–compost interactions.

Germination rates were calculated based on the number of seeds germinated and the biomasses of the germinated plantlets (as compared to the corresponding controls). It is seen that germination rates based on the number of seeds germinated have always been above 90%; meanwhile, those calculated based on biomasses could be found substantially lower, down to as low as 50%. This practically means that while the compost residue of the PBAT-based bioplastic would not significantly inhibit germination on the basis of seeds germinated, even if the compost was added to the potting soil at a ratio of 1:1, it would still affect germination by resulting in scrubby plantlets compared to the corresponding control. This alerts us that more sophisticated standard test procedures should be established for ecotoxicity assessment for rating germination inhibition.

## 4. Conclusions

The 90-day composting experiment aimed at investigating the degradation dynamics of PBAT-based bioplastic bags in industrial conditions concluded with several key findings. The findings not only underscore the potential of composting as a viable solution for biodegradable plastic waste management but also emphasize the importance of improving composting parameters to achieve optimal results. The evidence suggests that, under appropriate conditions, PBAT-based bioplastics can be effectively composted, thereby mitigating their environmental impact. However, the study also underscores the importance of ongoing monitoring and optimization.

The initial C/N ratio of 35.6 ± 5.3 was slightly above the maximum value of the literature recommendations. While the EN14045:2003 [29] standard suggests an optimal range for efficient composting, the experiment began with a C/N ratio outside this ideal range. Despite this, there was a substantial decline in the ratio, ending at 15.9 ± 0.3, which is closer to the values reported by Itävaara et al. (1997) [60]. Similarly, the MC showed a significant decrease from 46.08% ± 1.29 to 35.90% ± 1.3 during the high-temperature phase of the composting process. This rapid decline in moisture, coupled with sustained temperatures above 50 °C, likely led to the dehydration of the compost pile. Such dehydration could have hindered the proper degradation of materials, an aspect that should be carefully managed.

Although the degree of disintegration reached an impressive 95% ± 1.86 by the 12th week, the visual examination of the compost revealed a complex matrix. This complexity suggests that while disintegration was successful, the compost still contained intricate material structures that might need further degradation. From the FTIR and extract composition results, it can be concluded that compared with starch, PBAT does not fully degrade. The results of GC-MS analysis of the composted biopolymer indicated that PBAT has partially degraded to intermediate metabolites (4-hydroxybutyl)adipate (AA+), bis(4-hydroxybutyl)adipate (AA++), and (4-hydroxybutyl)terephalate (PTA+). Ecotoxicity tests showed interactions between compost and plant species. While some plants, such as Chinese cabbage, demonstrated positive results to the compost, others, like white mustard, displayed pronounced sensitivity. Such disparities underscore the importance of tailoring compost formulations based on the intended agricultural application. Morphological evaluations, both through optical microscopy and SEM, provided comprehensive insights into the intricate microstructural alterations bioplastics undergo during composting.

Chemical composition changes, as revealed through FTIR spectroscopy and GC-MS, validated the degradation pathways. The FTIR spectra provided insights into the biopolymer’s hydrolytic and enzymatic degradation, especially the breakdown of ester linkages within PBAT and the hydrolysis of starch. GC-MS analysis further corroborated these findings, with significant molecular shifts observed throughout the composting process. The experiment also revealed the presence and persistence of terephthalic acid in compost.

Based on the GC-MS results, we can conclude that the PBAT biopolymer is not fully decomposed. Even particles with a size above 2 mm are still present in the compost after the 12th week. As expected, the decomposition products appeared in the samples taken in the first period. In addition to monomers, more complex molecules (acid butanediol esters) have also been detected. These products did not disappear from the composted material after 1 year; thus, neither of them was a suitable marker to indicate the end of degradation. The observed differences among the parallel samples confirm that the rate of decomposition is strongly influenced by the local conditions in the compost, including the temperature. Under favorable conditions, the crystallization of amorphous PBAT polymer may lead to a more stable phase, which is prone to resist biodegradation. Disintegration of the biopolymers results in microplastic, which may remain in the compost for a longer period of time.

For future studies, it is recommended to focus not only on the general compost matrix but also on the finer fractions, particularly those less than 2 mm. Additionally, careful examination of microplastics in the compost should be conducted. This will ensure a comprehensive understanding of the degradation process and the environmental safety of the resulting compost. Moreover, it is crucial to optimize composting conditions, especially MC and temperature, to prevent dehydration and ensure effective degradation. The role of compost additives and the persistence of specific compounds like terephthalic acid warrant further investigation. Additionally, a more in-depth analysis of compost fractions and the potential generation of microplastics will be essential for assessing the environmental impact of composting bioplastics like the PBAT-based bioplastic studied. The results of the current study underline above all that decomposition of the PBAT polymer has not been completed during the study period, while according to the current guidelines, it could be considered decomposed based on both visual tools for mechanical fragmentation and germination tests. This indeed raises the need for more precautious governance and further assessment of the bioplastic decomposition process.

It would also be beneficial to explore the effects of different pre-treatment methods on the composting process. It would be worthwhile to establish a requirement for analyzing the compost for potentially toxic compounds and ensuring that it is safe for soil application in agricultural fields. Furthermore, understanding the long-term effects of compost derived from bioplastics on soil health, fertility, and crop yield is essential. For this reason, in composting plants where biopolymers are also treated, the examination of microplastics must be recommended during the authorization procedure. However, challenges in measuring microplastics in a solid matrix and the shortcomings of current standards need to be assessed. This work can significantly increase our understanding of the intricate microplastic–compost interactions and highlights the potential analytical methods of quantification of said components. To conclude, studying the composability of bioplastics under industrial conditions is vital because, as the world grapples with plastic pollution, bioplastics present a sustainable alternative. Ensuring their safe and efficient composting in industrial settings paves the way for a circular economy, where materials are continually reused and recycled, minimizing the environmental impact.

## Figures and Tables

**Table 1:** Overview of the published standards for biodegradation of biopolymers under aerobic biological treatment environments.

Standard	Name	Geographical Validity
EN 13432:2001 [26]	Packaging. Requirements for packaging recoverable through composting and biodegradation. Test scheme and evaluation criteria for the final acceptance of packaging	European Union
EN 17033:2018 [27]	Plastics—Biodegradable mulch films for use in agriculture and horticulture—Requirements and test methods	
EN 14995:2006 [28]	Plastics—Evaluation of compostability—Test scheme and specifications	
EN 14045:2003 [29]	Packaging—Evaluation of the disintegration of packaging materials in practical oriented tests under defined composting conditions	
EN 14806:2005 [30]	Packaging—Preliminary evaluation of the disintegration of packaging materials under simulated composting conditions in a laboratory-scale test	
ISO 17088:2021 [31]	Plastics—Organic recycling—Specifications for compostable plastics	Worldwide
ISO 16929:2021 [32]	Plastics—Determination of the degree of disintegration of plastic materials under defined composting conditions in a pilot-scale test	
ISO 18606:2013 [33]	Packaging and the environment—Organic recycling	
ISO 20200:2023 [34]	Plastics—Determination of the degree of disintegration of plastic materials under simulated composting conditions in a laboratory-scale test	
ISO 17556:2019 [35]	Plastics—Determination of the ultimate aerobic biodegradability in soil by measuring the oxygen demand in a respirometer or the amount of carbon dioxide evolved	
ASTM D6400:2022 [36]	Standard Specification for Labeling of Plastics Designed to be Aerobically Composted in Municipal or Industrial Facilities	USA
ASTM D6868:2021 [37]	Standard Specification for Labeling of End Items that Incorporate Plastics and Polymers as Coatings or Additives with Paper and Other Substrates Designed to be Aerobically Composted in Municipal or Industrial Facilities	
AS 43736:2006 [38]	Biodegradable Plastic—Biodegradable Plastics Suitable for Composting and other Microbial Treatment	Australia

**Table 2:** Characteristics of the studied biopolymer.

Characteristic	Value	Test Standard
Melting Temperature	110 °C	ASTM D3418 [43]
Density	1270 kg m^−3^	ASTM D792 [44]
Tensile Modulus	350 MPa	ASTM D882 [45]
Elongation at Break	230%	ASTM D882 [45]

**Table 3:** Measured parameters during the study.

Measured Parameter	Test Method
Carbon-to-nitrogen ratio of the input material (C/N)	Elemental analysis to measure the carbon and nitrogen contents of the initial mixture MSZ EN ISO 16948:2015 [49], MSZ EN ISO 16994:2017 [50]
Organic matter (OM)	Loss on ignition (MSZ EN 15935:2012) [51]
Moisture content (MC)	EN 16086-1:2012 [52]
pH	Potentiometric determination of H^+^ ion concentration, MSZ EN 13037:2012 [53]
Electrical conductivity	ISO 7888:1985 [54]
Salt content	ISO 8502-6:2020 [55]
Bulk density	ISO 8502-9:2020 [56]
Respiration intensity	Malinska, K. (2016) [57]

**Table 4:** Organic matter (OM) and moisture content (MC) during the experiment.

Composting Week	OM (%)	MC (%)
0	77.43 ± 9.47	46.08 ± 1.29
2	74.09 ± 6.77	41.46 ± 0.17
4	75.60 ± 1.46	41.15 ± 1.20
6	73.74 ± 8.18	46.42 ± 1.25
8	69.79 ± 1.32	40.81 ± 0.02
10	65.07 ± 3.81	36.97 ± 0.77
12	62.43 ± 1.25	35.90 ± 1.36
M1 ^a^	60.04 ± 1.12	5.46 ± 1.21

^a^ 1-year matured compost.

**Table 5:** Summary of wavenumbers and their corresponding functional groups.

Peak Wavenumbers in Current Study (cm^−1^)	Vibration	Peak Wavenumbers from Scientific Literature (cm^−1^)	Functional Group Assigned	Material	Reference
725	Asym. deformation	726	–CH_2_–	PBAT	[40]
1025	Deformation	1016	Phenyl ring	PBAT	[75]
1110	Stretching (alcohols)	1104	C–O	PBAT	[75]
1274	Stretching (ester)	1268	C-O in the ester	PBAT	[40,79]
1410	CH_2_CO sym. deform.	1409	C(–H)_2_	PBAT	[73]
1450	Stretching	1456	Phenylene group	PBAT	[73]
-	-	1505	Benzene ring	PBAT	[75]
1720	Stretching	1710	C=O	PBAT	[40,75]
1270 (shoulder)	Stretching	1270	C–O	Starch	[73]
-	-	1445–1225	C–H	Starch	[80]
3300	Stretching	3900–3300	O–H	Starch	[78]
2874, 2957	Sym./asym. stretching	2920	C–H	Starch	[78]
-	-	1250–900	C–O	Starch	[78]
1150	Shoulder	1164	CH_2_OH	Starch	[73]
1085	Shoulder	1081	C–O	Starch	[73]

**Table 6:** Effects of the composted biopolymer on germination dynamics determined in standard test protocols using common indicator plant organisms.

Test Species	Treatment ^a^	Duration	No. of Seeds	Seeds Germinated	Biomass	Germination Rate		Test Standard
						by Seed Number	by Biomass	
		(Day)	(pc)		(g)	(%)		
Chinese cabbage (*Brassica rapa* ssp. *pekinensis*)	C	5 and 37	20	18.25 ± 2.36	65.37 ± 6.47			EN 16086-1 [52]
	T			16.50 ± 1.29	60.94 ± 17.78	90.4	93.4	
Spring barley (*Hordeum vulgare*)	C	5 and 16	20	18.75 ± 0.96	12.48 ± 0.48			EN 16086-1 [52]
	T			18.50 ± 1.91	8.38 ± 0.59	98.7	67.2	
White mustard (*Sinapis alba*)	C	16	25	25	10.91 ± 0.87			MSZ 08-0012-4:1979 [58] ^b^
	T			25	5.44 ± 0.37	100	49.9	

^a^ C: control experiments carried out in potting soil; T: treatment experiments carried out in compost mixed at a 50:50 ratio with potting soil; ^b^ discontinued national test standard.

## Data Availability

Data are contained within the article.
